# Smart Bioinks as de novo Building Blocks to Bioengineer Living Tissues

**DOI:** 10.3390/gels5020029

**Published:** 2019-05-22

**Authors:** Andreas Blaeser, Sarah C. Heilshorn, Daniela F. Duarte Campos

**Affiliations:** 1 Medical Textiles and Biofabrication, RWTH Aachen University, 52074 Aachen, Germany; andreas.blaeser@ita.rwth-aachen.de; 2 Department of Materials Science & Engineering, Stanford University, Stanford, CA 94305, USA; heilshorn@stanford.edu

## 1. Introduction

In vitro tissues and 3D in vitro models have come of age [1,2,3]. Engineered tissues can be used to substitute failed ones, such as skin [4] or cartilage [5], or as biotech microchips to predict the efficacy of new drugs against diseases, such as cancer [6,7]. In vitro models, fabricated by bioprinting or other additive manufacturing strategies, are valuable alternatives to state-of-the-art pre-clinical animal experiments, because they can be produced with patient-specific cells or humanized synthetic cells containing patient-specific genetic information. In vitro human models have two key advantages: (1) Animal experiments can be replaced, which is in accordance with global ethical guidelines and legal requirements proposed by current European regulations, and (2) in vitro human models may provide more accurate predictive values of actual human biology compared to animal models. 

Hydrogels (often simply called gels) with embedded cells are the gold-standard materials used as bioinks in bioprinting [8,9]. Gels provide physical, chemical, and biological support to cells, which are embedded in the gels before, during, and after bioprinting. The use of compatible gels in bioprinting is essential for obtaining functional 3D tissues and must be viewed holistically to include biological, physical, and chemical perspectives. The design of a gel as bioink for bioprinting can be challenging and highly dependent on the intended tissue application (skeletal, gastro-intestinal, cardiovascular, neural, cancer, etc.).

Gels used in bioprinting can be of natural or synthetic origin [10,11]. Natural gels, such as collagen-based materials, are cell-compatible solutions for mimicking tissues containing collagen like cartilage, bone, skin, etc [12]. Fibrin-based gels have been extensively used for cardiovascular applications [13]. Recombinant elastin-like gels have been employed for cartilage and neural tissue engineering [14,15]. Interestingly, gels composed of synthetic polymers, such as polyethylene glycol (PEG) and poly-lactic acid (PLA), have also been used to create engineered mimics of many of these same tissue types, despite their different structural and chemical properties, compared to natural gels [16,17].

**Figure 1:**
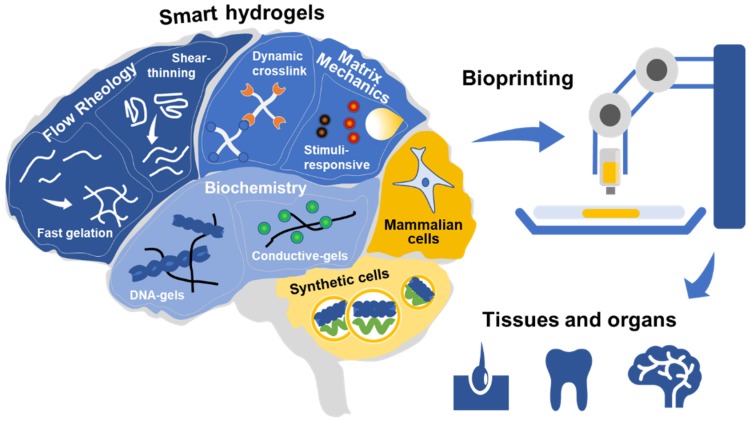
Smart hydrogels suitable for bioprinting to bioengineer tissues and organs. The flow rheology, matrix mechanics, and matrix biochemistry of the bioinks can be tuned to mimic the desired characteristics of native tissue. Human cells or humanized synthetic protocells contribute to making 3D bioprinted tissues living and functional.

There are three fundamental aspects to be considered when designing smart bioinks to bioengineer in vitro tissues: Flow rheology, final matrix mechanics, and matrix biochemistry (Figure 1). Whereas the rheological properties of a bioink during flow are selected to be convenient before and during bioprinting, the final matrix mechanics and biochemistry are of critical importance to regulate cell phenotype after bioprinting. The ability to tweak and tune each one of these material properties is what ultimately defines the potential applications of each bioink. Recently, complex bioink formulations, comprising mixtures of natural and synthetic monomers, as well as functional additives, have been reported in an attempt to match the initial rheological, final mechanical, and biochemical demands of 3D bioprinting [8,18,19]. Although these gels provide excellent short-term results, it is unclear if they will allow long-term cellular adaptation and tissue maturation, such as in a living tissue. In the future, the incorporation of so-called “smart” material properties into bioinks may significantly improve the maturation and functionality of engineered tissues, by allowing the material to dynamically alter its properties over time to meet the changing needs of the embedded cells. “Smart” materials are defined as those whose properties can undergo significant changes in response to relatively small environmental triggers.

## 2. Tweaking the Flow Rheology

The rheological properties and gelation kinetics of a bioink determine its flow behavior, its sol-gel transition, and its viscoelastic response [20,21,22]. Controlling these factors is critical in reducing printing-related cell damage and enabling high printing precision [23]. Thus, thorough characterization of these properties and the ability to tweak them is highly desirable for robust bioinks. As an example, it is well known that the viscosity of a bioink can be altered by varying its monomer concentration or supplementing with additives. An ideal bioink must be engineered to have appropriate flow rheology, both during the printing process and immediately post-printing. In the future, this may require two distinct stages of rheological properties. For example, bioinks that shear-thin during the printing process, such as gelatin methacryloyl [24] and nanosilicate-loaded kappa-carrageenan [25], are developed for improving printability, shape retention, and cell viability during bioprinting. Hydrogels displaying many different types of non-Newtonian behavior (e.g., pseudoelastic, dilatant, and Bingham rheology) are routinely applied. Versatile bioink gelation mechanisms, including both physical (e.g., ionic/electrostatic/hydrophobic interactions, hydrogen bonding, or crystallization) and chemical (e.g., covalent bonding such as “click chemistry” and photopolymerization) crosslinking strategies have been developed [26,27]. Immediately post-printing, the bioink could be designed to have rapid gelation kinetics to improve the shape fidelity, as has been demonstrated by bioinks with enzymatic [28] and electrostatic [29] pre-crosslinking. Moreover, single- and dual-stage crosslinking bioinks, with distinct rheological properties during and after bioprinting, have shown both shear-thinning and self-healing behaviors, either via guest–host bonding with improved stabilization using photopolymerization (dual-stage crosslinking [30]) or oxidized alginate with imine type crosslinks (single-stage crosslinking [31]).

In the future, smart hydrogels could be designed to not only allow printability, but to enable reversible switching of rheological properties. This type of exquisite control over flow rheology may be useful, for example, when printing complex, hierarchical structures that require different levels of print resolution, and hence different ink viscosities, in different regions of the printed construct. Recent studies have demonstrated the feasibility of this idea. For example, Accardo and Kalow developed a dynamic hydrogel with reversible viscoelasticity by irradiating hydrogels bearing azobenzene-boronic acid and diol end groups with two different wavelengths of light [32]. Chu and Feng reported smart micelle fluids, which exhibit stimuli-responsive alterations of their viscosity profile [33]. Although these specific materials have not yet been demonstrated to be cell-compatible, their design strategies suggest that similar concepts could be transferred to smart bioink development.

## 3. Tuning the Final Matrix Mechanics

Following bioprinting, cell-containing bioinks are cultured for several days or weeks with the goal to fabricate living tissue [34,35]. Many groups have demonstrated that the mechanics of the cellular microenvironment can impact several cell behaviors, including proliferation, migration, differentiation, and fate [36]. While this observation was originally reported for elastic-like gels displaying a time-independent stiffness, it is now known that time-dependent mechanical properties (i.e. viscoelasticity) also impact cell behavior [37,38,39]. Hydrogels that provide poor or contradicting mechanical signals, with respect to cell function over time, may demonstrate successful short-term results (e.g., cell encapsulation) but may not support long-term transformation towards the desired tissue or organ mimic. A common dilemma in these systems is that it can be challenging to engineer a material that has the necessary biologically relevant stiffness without inhibiting cell spreading and migration. Adaptable hydrogels offer a potential solution to this conflict of objectives. Adaptable hydrogels are networks that can reversibly crosslink, giving cells the freedom to spread, migrate, and proliferate without degrading the material. For example, many types of dynamic covalent bonds have been used to crosslink biopolymers, including PEG and proteins [40]. Encapsulated cells in these dynamic hydrogels often show varying morphological changes, depending on the rate of stress relaxation [40]. This idea has recently been demonstrated in a printable bioink that uses dynamic covalent bonds to alter the matrix stress relaxation rate and hence the amount of cell spreading [31]. This approach may be ideal for designing bioprinted tissues, although further development will be necessary to ensure the 3D-printed shapes can be maintained for longer incubation periods.

Besides structural integrity and cell fate determination, bioink mechanics play an important role in dynamic conditioning of tissues. In response to external forces exerted in shearing [41], compressing [42], or stretching bioreactors [43], biomimetic tissue formation can be observed. In the future, smart bioinks may enable more efficient application of mechanical stimulation to encapsulated cells. Local alteration of the gel mechanical properties could be used to exert forces onto cells. This can be achieved, for instance, by integration of light, electric, or magnetic responsive particles or polymers into bioinks [44,45,46,47]. 

It should also be noted that several bioinks currently in use do not allow independent variation of both the matrix mechanical and biochemical properties. For example, when polymer concentration is used to increase the matrix biomechanics, this also increases the concentration of cell-adhesive binding ligands, altering cell biochemistry. Similarly, some bioinks use the addition of fillers, such as nanocellulose [48] or nanoclay [49,50] to increase matrix mechanics. However, these changes directly affect the porosity and topographical structure of the network, thereby altering the local presentation of cell-adhesive binding ligands, which affects cell adhesion, migration, and proliferation potential. Fortunately, many examples exist in the broader biomaterials literature where innovative materials have been designed to decouple these two matrix properties, and these design strategies could be further adopted by the bioprinting community. Accordingly, one area of promise for future bioink development is the design of smart materials that enable the decoupled tuning of ink mechanics and biochemistry. Chemical modification of polysaccharide backbones, e.g., by carboxylation [18,51,52] or sequential hydrogel crosslinking [53], are recent examples, showing how this goal could be achieved.

## 4. Engineering the Biochemistry

Biocompatibility, molecular recognition, and functionality are the properties that confer biomimicry to 3D bioengineered tissues. As a minimum requirement, all reagents and materials used during bioprinting should be biocompatible. Importantly, some reagents, such as photoinitiators, may be cell compatible at shorter times, but toxic to cells at longer times. Thus, the choice of bioink chemistry can restrict the printing process. Several studies have demonstrated approaches to integrate native elements of molecular recognition into 3D hydrogel cultures, which can be directly transferred and applied to bioinks. A common strategy is to include peptide and protein molecules found in the natural extracellular matrix into engineered bioinks. These molecular recognition motifs enable cell-surface receptors to bind to the bioink, enabling cell-detection of matrix mechanics. A newer concept is the incorporation of DNA molecules into a bioink, either as the polymer backbone or a cross-linker that connects the main building blocks [54]. DNA-derived bioinks can be designed with stimuli-responsive properties in order to tune the rheological, final mechanical, and biochemical characteristics of the material. For example, a recent study presented a DNA hydrogel capable of producing RNA and interfering with protein expression [55]. Cells encapsulated in this gel were able to internalize and engulf the DNA gel by receptor-mediated endocytosis. Prospectively, DNA can be synthesized into specifically designed sequences for the 3D printing of personalized implants for therapeutic gene editing [56]. 

Besides molecular recognition, other material functionality can play an important role in mimicking artificial tissues. For example, in cardiovascular and neural research it may be necessary to consider the electroconductivity of the chosen bioink. In line with this, electroconductive hydrogels with functional pendant groups have been synthesized, showing responsive electroactivity in physiological conditions [50,57]. These functionally smart hydrogels have been used as cardiac patches in preliminary in vivo studies, showing their compatibility with the rhythmogenic activity of the heart [58].

## 5. The Last Piece of the Puzzle: Living Cells for Living Tissues

Bioprinting is defined as the manufacturing of complex 3D constructs, using a combination of living cells and biomaterial inks. The living cell could be either of human or animal (murine, ovine, canine, etc.) origination. Logically, bioengineered human tissues and organs are valuable for human regenerative medicine, whereas animal-based tissues are relevant in veterinary medicine and as models of human disease. Attempts to fabricate human blood vessels, heart, liver, cornea, and cartilage were done using human primary endothelial cells [59], cardiomyocytes [60], hepatocytes [61], keratocytes [62], and chondrocytes [63]. Besides primary adult cells, stem cells [64,65], pre-differentiated cells [14], and organoids [66,67,68] were used for bioprinting applications and have great potential.

Considering the use of bioprinted in vitro models as diagnostic tools, one could consider advancing from traditionally utilized mammalian cells to synthetic biology. New-generation diagnostic tools could potentially provide new ways to dynamically probe and monitor human pathobiology. In line with this, and as an exciting alternative to mammalian cells, synthetic protocells could potentially be encapsulated into smart bioinks as a replacement to human and animal cells to develop complex 3D artificial tissues. An artificial protocell is a synthetic, cell-like structure, consisting of a phospholipid bilayer membrane that encapsulates molecular components found in natural cells, such as synthetic DNA and enzymatic machines. Artificial cells can be synthesized using only non-living components. Recently, artificial protocells were synthesized with enough genetic information to self-replicate and maintain some basic cellular functions, such as molecular self-organization [69,70]. In the future, synthetic biology morphogenetic mechanisms, such as patterning by phase separation, may allow the construction of an artificial “developmental cycle” by invoking cell proliferation, fusion, migration, and differentiation through changes in gene expression [71]. In one potential application, artificial tissues could sense and adapt to their surroundings, and, thus, deliver drugs more precisely to their targets. Although still an emerging technology, synthetic cell biology offers tremendous potential for use in 3D bioprinting of tissues.

## 6. Concluding Remarks

Research on cell patterning and bioprinting dates to the beginning of the 21^st^ century. Technological questions led the debate in the early stages of bioprinting research. Is it feasible to print living cells? What are the boundary conditions for a safe cell-substrate transfer? Which methods can be used to assemble cells and biomaterials (e.g., microextrusion, inkjet, laser-assisted)? In parallel, research groups began to shed light on basic biomaterial questions. What environments enable 3D cell culture? Is it possible to design 3D-bioprintable materials without causing cell damage? Is it possible to modulate viscoelasticity in order to favor cell-matrix interactions? These requirements for the ink material continue to increase over time, and gels that simply encapsulate living cells and are permissive of bioprinting will be substituted by bioinks that support short-term and long-term cellular demands.

Within the last two decades, tremendous advances have been made in the development of 3D bioprinting, and many materials that are both cell-compatible and printable have been reported. Hydrogels that meet these two key requirements are now considered to be excellent bioinks; however, future development of smart bioinks is poised to push the field further. Besides simply providing the necessary flow rheology, final matrix mechanics, and matrix biochemistry to achieve viable cell culture and printability, smart bioinks will enable dynamic manipulation of these terms. Smart hydrogels can potentially interact with living organisms through reciprocal feedback, where the hydrogel instructs the organism and the organism causes the hydrogel to adapt. Thus, future bioinks may be able to adapt and “mature” along with the maturation of encapsulated cells to form truly functional tissue. Combining dynamic control of hydrogel mechanics and biochemistry with the advancing research on synthetic biology, artificial tissues created using smart bioinks will form a new class of person-made tissues. Projecting this thought into the future, visionary smart bioinks will provide a symbiotic environment for dynamic, responsive interactions between the synthetic matrix and the encapsulated cells. In these newly bioinspired ecosystems, the roles and tasks of cells and biomaterials will be newly defined.
